# The effects of lumbar fusion and non-fusion surgery on the development of Modic changes

**DOI:** 10.1186/s13018-022-02971-3

**Published:** 2022-07-01

**Authors:** Xiaoping Mu, Seong Woong Kim, Eberhard Uhl, Karsten Schöller

**Affiliations:** 1 Department of Spine Surgery, The People’s Hospital of Guangxi Zhuang Autonomous Region, Guangxi Academy of Medical Sciences, No. 6, Taoyuan Road, Nanning, 530021 Guangxi China; 2 Department of Neurosurgery, Justus-Liebig-University, Giessen, Germany; 3 Clinic for Spinal Surgery, Schoen Clinic Hamburg Eilbek, Hamburg, Germany

**Keywords:** Modic changes, Lumbar degenerative disease, Lumbar fusion, Discectomy, Sequestrectomy, Magnetic resonance imaging

## Abstract

**Background:**

The aim of this study was to investigate the influence of lumbar fusion and non-fusion surgery on the postoperative development of Modic changes (MCs).

**Methods:**

A total of 270 patients who underwent lumbar fusion, microsequestrectomy, microdiscectomy, and microdecompression, and who were examined by pre- and postoperative magnetic resonance imaging during the period of January 2012 to December 2018, were included in this retrospective study. The incidence of new postoperative MCs and the change of volume of preexisting MCs after surgery were investigated.

**Results:**

The total incidence of new MCs following lumbar surgical procedures was 36.3%. Lumbar fusion showed a tendency towards a lower postoperative incidence of new MCs than the other three lumbar surgical procedures. The first postoperative year seems to be the most active phase for the development of new MCs. The postoperative volumes of MCs in patients who underwent lumbar non-fusion procedures were significantly greater than those before surgery (*P* < 0.01). However, no significant difference was detected between pre- and postoperative volumes of MCs in patients with lumbar fusion (*P* > 0.05).

**Conclusion:**

Lumbar surgical procedures contribute to the development of new MCs, particularly non-fusion surgeries. However, further studies are needed to confirm the clinical relevance of these findings.

## Background

Since the classification of vertebral endplate signal changes on magnetic resonance imaging (MRI), also known as Modic changes (MCs), and since histological features had been described systematically by Modic et al. [1, 2], the clinical relevance and interpretation have become current research hotspots. Up to date, the aetiologies and pathogeneses of MCs have remained unclear. However, many studies suggested that a variety of factors work together to contribute to the occurrence of MCs. Abnormal gene fragments may be the basis of the occurrence of MCs [3, 4]. Autoimmune reactions and inflammation induced by inflammatory mediators and extracellular matrix catabolites may gradually aggravate both the extent of disc degeneration (DD) and vertebral body bone marrow lesions [5, 6]. Furthermore, a previous study has shown that the endplate and intervertebral disc damage caused by biomechanical imbalance might provide favourable conditions for immune responses [7]. Therefore, a biomechanical factor also has to be considered in the process of the development of MCs.

For patients with lumbar degenerative disease, several common surgeries including discectomy, sequestrectomy, decompression, and fusion are currently proven safe and effective. However, these surgical procedures seem to directly alter the biomechanical forces on a microstructural level and predispose the adjacent vertebral bodies to MCs [8], especially in the case of a disrupted disc/an injured annulus fibrosus.

The influence of different surgical techniques on endplate changes may be different [6]. Previous studies [9, 10] have reported that there were increases in the prevalence and sizes of MCs following lumbar discectomy. However, studies investigating the influences of several common lumbar surgical procedures on the development of new MCs are still lacking. Additionally, the methods used by previous studies to measure the sizes of MCs were qualitative or semi-quantitative analyses, which might have great errors for irregular shapes of MCs. Therefore, the purpose of the present work was to investigate the influence of lumbar fusion and non-fusion surgery (microsequestrectomy, microdiscectomy, and microdecompression) on the postoperative development of MCs by combining a qualitative and three-dimensional quantitative analysis.

## Materials and methods

### Study population

In this retrospective study, we collected the data of 270 subjects (≥ 18 years of age) with lumbar degenerative disease treated in the period of January 2012 to December 2018 at Justus-Liebig-University Giessen Medical Center based on an electronic inpatient database. We implemented this study in strict accordance with the principles of the Helsinki Declaration, and the study protocol was approved by the ethical committee of Justus-Liebig-University Giessen (No. AZ139/20).

Adult patients who met the following criteria were included in this study: (i) persisted chronic low back pain more than 6 months; (1) met the surgical indicators and underwent one of the following surgical procedures: microdiscectomy, microsequestrectomy, microdecompression (unilateral laminotomy for bilateral decompression; ULBD), instrumented Transforaminal Lumbar Interbody Fusion (TLIF); (ii) had both a preoperative and postoperative lumbar MRI. We excluded those patients who (1) had a previous history of lumbar surgery; (2) took some medicine (calcitonin, and bisphosphonate) that might influence the progression of MCs before and/or after surgery; (3) had infectious or inflammatory diseases of spine.

### Magnetic resonance imaging

The patients lying in the supine position were examined with 3.0 T (Skyra, Siemens, Germany) or 1.5 T magnetic resonance units (Espree, Siemens, Germany). The scanning range was from T12 to sacrum. All images were evaluated by spinal surgeons with more than three-year clinical experience (XM, SK). The dispute regarding evaluation results was resolved by consulting an extra reviewer with more than ten-year clinical experience (EU, KS).

The following parameters were applied (1) with 3.0 T unit: the sagittal T1-weighted images (T1WI) (slice thickness (ST): 3 mm, time of repetition (TR): 650 ms, time to echo (TE): 9.9 ms, field of view (FOV): 280 mm), the sagittal T2-weighted images (T2WI) (ST: 3 mm, TR: 3000 ms, TE: 102 ms, FOV: 280 mm); and the axial T2WI (ST: 3 mm, TR: 3500 ms, TE: 108 ms, FOV: 210 mm); (2) with 1.5 T unit: the sagittal T1WI (ST: 3 mm, TR: 620 ms, TE: 13 ms, FOV: 280 mm), the sagittal T2WI (ST: 3 mm, TR: 4850 ms, TE: 81 ms, FOV: 280 mm); and the axial T2WI (ST: 3 mm, TR: 5000 ms, TE: 97 ms, FOV: 220 mm).

### Evaluating Modic changes

**Fig. 1:**
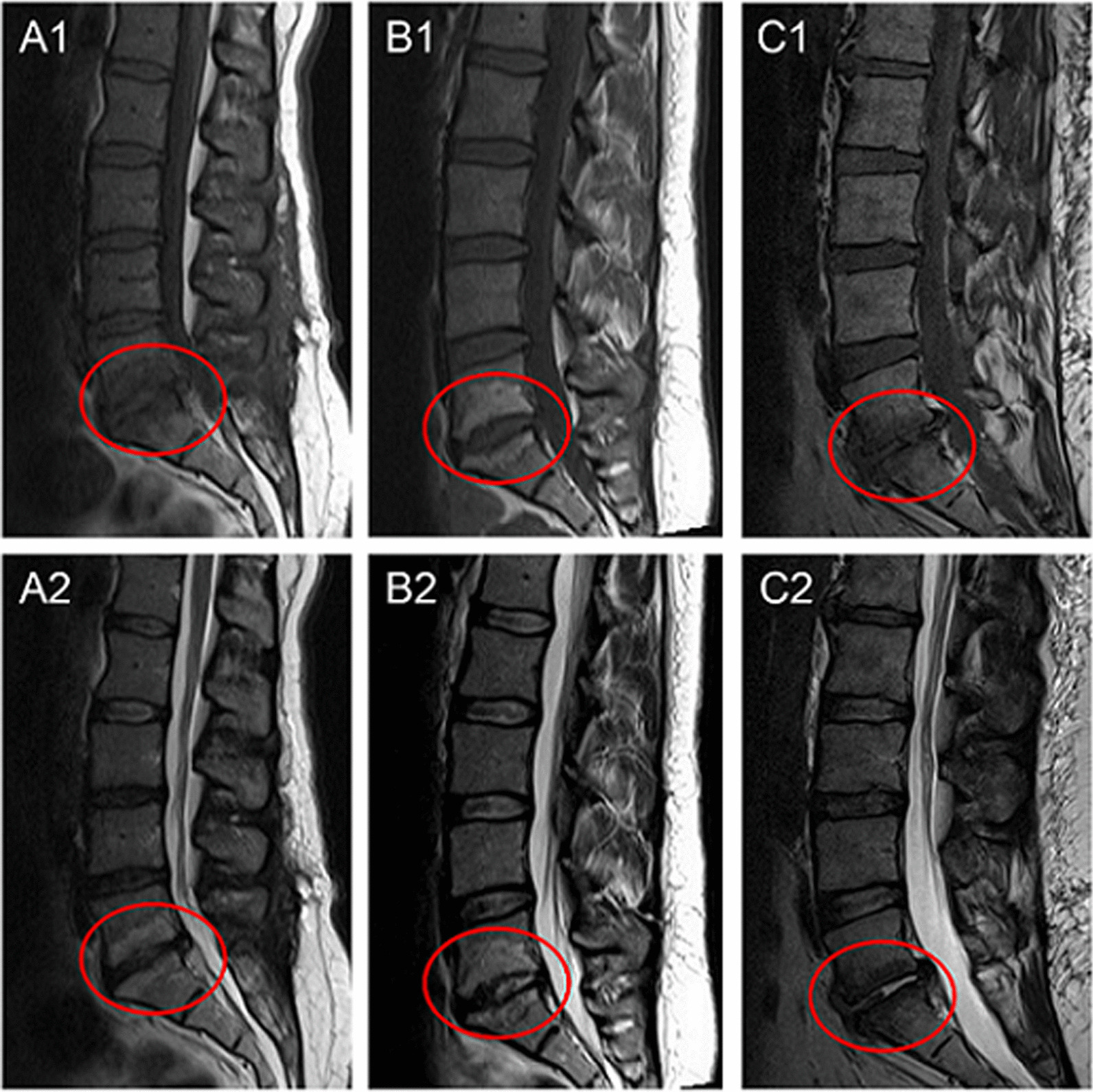
Appearance of three types of Modic changes. Modic type 1 change (MC1): hypointense on T1WI (**A1**) and hyperintense on T2WI (**A2**); Modic type 2 change (MC2): hyperintense on both T1WI (**B1**) and T2WI (**B2**); Modic type 3 change (MC3): hypointense on both T1WI (**C1**) and T2WI (**C2**)

Three inter-convertible types of MCs have been described based on their appearance in T1- and T2-weighted MR images [1, 2]. Modic type 1 change (MC1) shows decreased signal intensity on T1WI and increased signal intensity on T2WI; Modic type 2 change (MC2) demonstrates increased signal intensity on both T1WI and T2WI, whereas Modic type 3 change (MC3) reflects decreased signal intensity on both T1WI and T2WI (Fig. 1). For some patients with mixed MCs, those with a hypointensity on MRI (bone marrow oedema) were classified into MC1 while those mainly with a hypersignal (fat degeneration) into MC2 [11].

### Counting and definition of new MCs

**Fig. 2:**
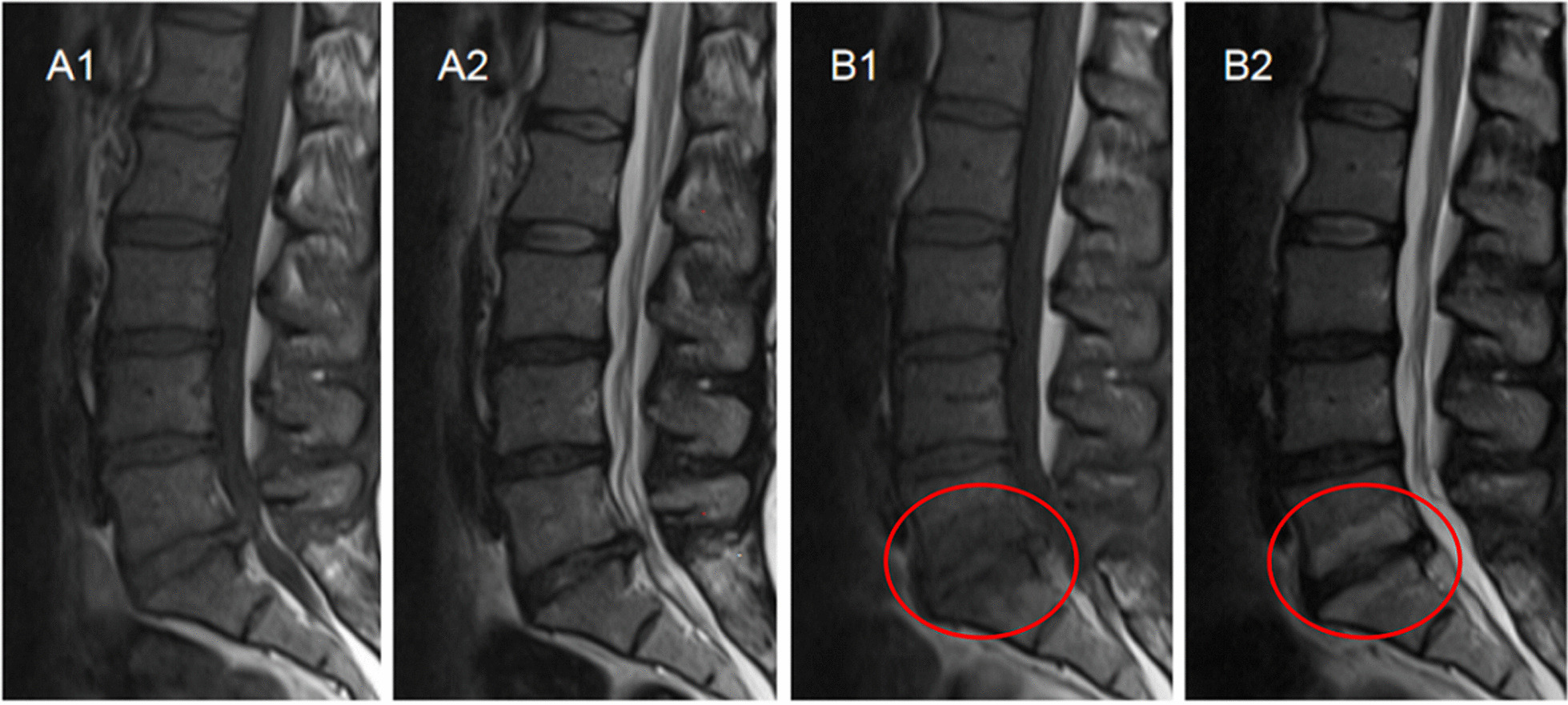

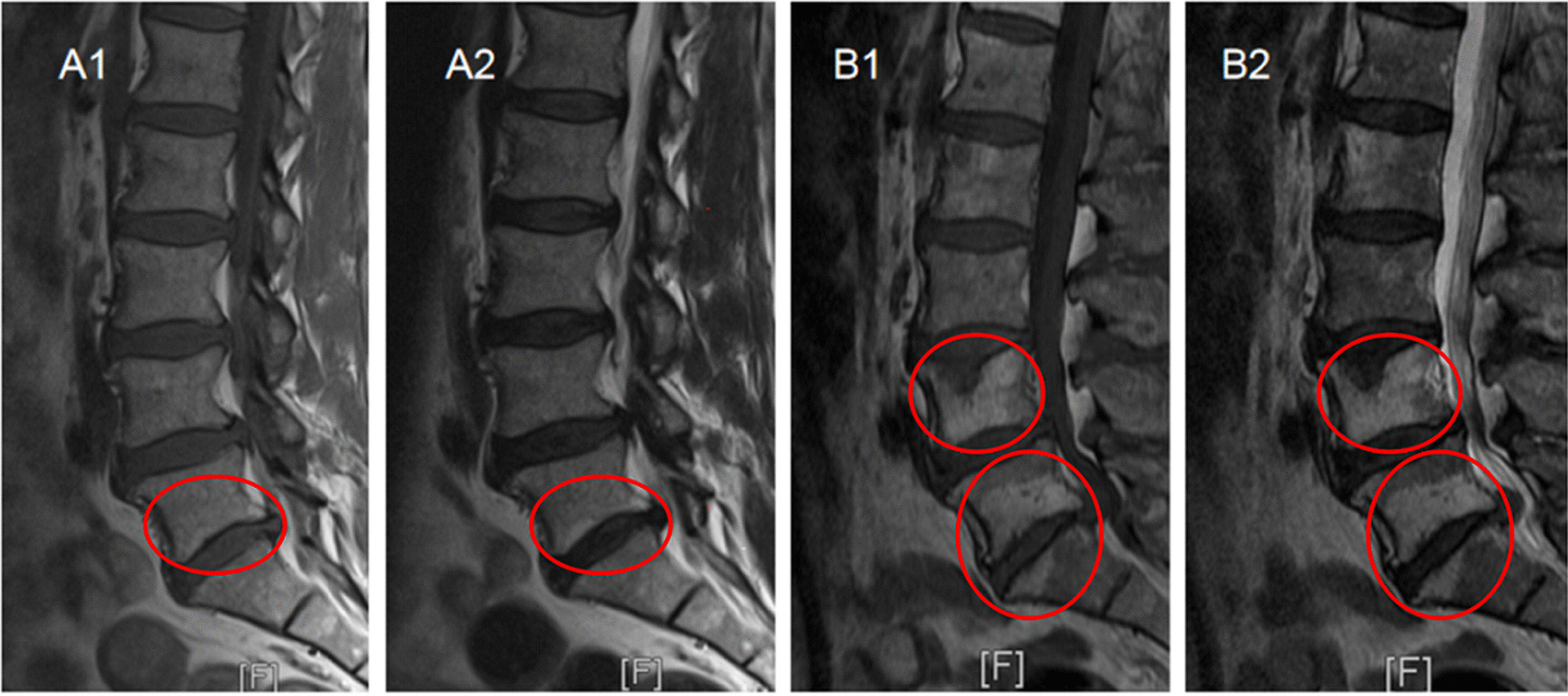
**a** Definition of new Modic changes (MCs) (1). No MCs at both the surgical and their adjacent levels before surgery (**A1, A2**), new MCs at the surgical level after surgery (**B1, B2**). **b** Definition of new Modic changes (2). Modic type 2 change (MC2) at the surgical level before surgery (**A1, A2**), new MC2 at its adjacent level (**B1**) and the volume of preexisting MC2 at the level of L5/S1 increased after surgery (**B2**)

In this study, only MCs that occurred at the surgical levels and/or their adjacent levels were counted. The lumbar segments with one of the following conditions were defined as new MCs: (1) The preoperative MRI scan revealed no MCs at both the surgical and their adjacent levels, but there were new MCs at the surgical or their adjacent levels after surgery (Fig. 2a). (2) The surgical (or adjacent) levels had evidence of MCs on the preoperative MRI and new MCs were observed in their adjacent (or surgical) levels at follow-up (Fig. 2b).

### Calculating the volumes of MCs

The platform-like volume calculation formula proposed by Wang et al. [12] was used to measure the volumes of MCs because of the irregular shapes of MCs. This method was based on the Cavalieri principle in stereology: according to the method of isometric sampling, the total area of all sections of the object is multiplied by the cross section spacing in any direction, that is, the unbiased estimation of the volume of the object.

**Fig. 3:**
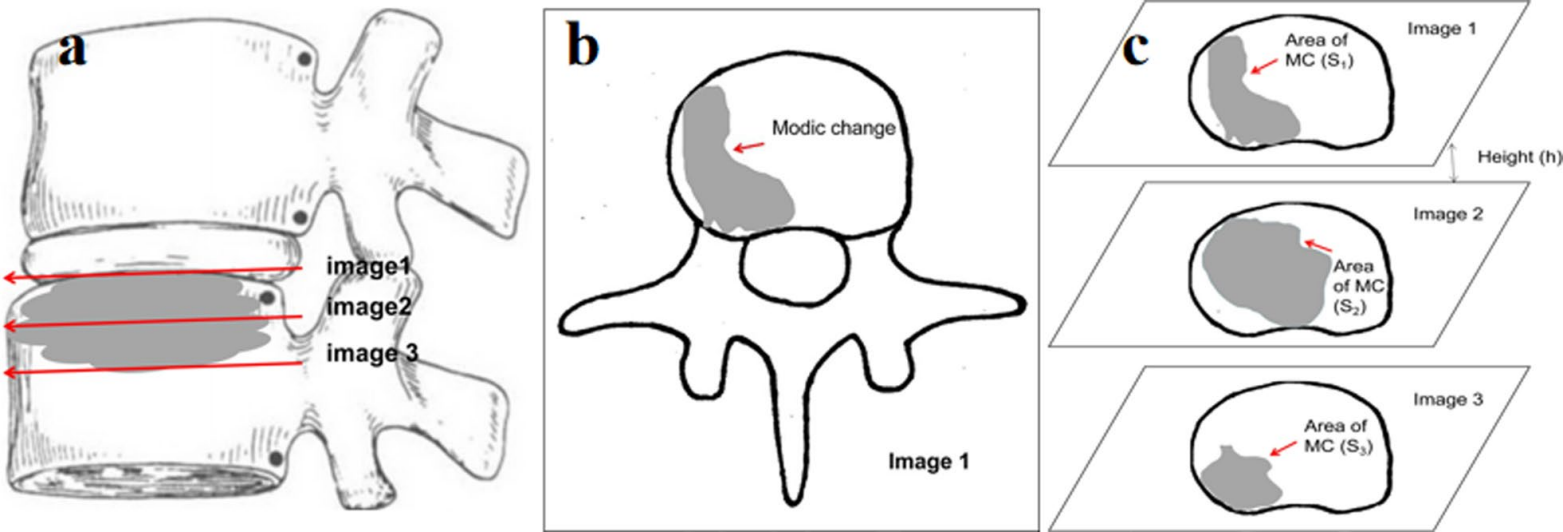
Measuring the volume of Modic changes on magnetic resonance imaging (MRI).** a** Dividing MCs in each lumbar level into several scanning layers (image 1, 2, and 3);** b** The area of MC in the horizontal MR images;** c** Schematic diagram of volume calculation for MC:  *V* = (*S*_1_ + 2*S*_2_ + *S*_3_) *height/2

The MCs in each lumbar level were divided into multiple scanning layers after MRI scanning, with the interval between two scanning layers remaining the same. Layers on horizontal MR images that contained MCs were selected. The boundary between the MCs and normal vertebral tissues in each scanning layer was delineated using the paintbrush in the medical imaging system to automatically calculate the area of the MCs in this layer. The section of MCs between two scanning layers in the MRI was regarded as an independent platform. The volume of each platform was calculated using the formula: *v* = (*S*_1_ + *S*_2_)*h/2, in which *S*_1_ and *S*_2_ are the areas of the upper layer and lower layer of a platform, respectively, and *h* is the distance between two layers. The total volume of MCs was calculated by summing up the volumes of multiple platforms: *V* = (*S*_1_ + 2*S*_2_ + 2*S*_3_ + ·  + 2*S*_*n−*1_ + *S*_*n*_) *h/2 (Fig. 3).

### Statistical analysis

We used SPSS software (version 22.0, SPSS Inc., Chicago, Illinois, USA) to analyse the data. Percentage (%) was used to describe the prevalence of new MCs. Continuous data, such as the volumes of MCs, are expressed as the mean ± standard deviation (*x* ± *s*). For normally distributed continuous data, we used the paired sample test to compare the difference between preoperative and postoperative volumes of MCs. The Chi-square test was used to compare categorical data, such as the incidence of new MCs between the groups. A *P* value with two tails less than 0.05 was considered statistically significant.

## Results

A total of 270 patients were included in this study. Baseline characteristics of the patients are shown in Table 1.

**Table 1:** Baseline characteristics of the patients

Number of patients	*n* = 270
Gender	male: 130 female: 140
Age (years)*	62.8 ± 15.7
BMI (kg/m^2^)*	30.0 ± 5.9
CRP (mg/L)*	7.3 ± 17.6
Leukocyte count (G/L)*	8.5 ± 3.2
Follow-up time (days)*	267.6 ± 427.8
Type of surgery	Microdiscectomy (*n* = 93, 34.5%)
	Microsequestrectomy (*n* = 60, 22.2%)
	Microdecompression (*n* = 74, 27.4%)
	Fusion (TLIF) (*n* = 43, 15.9%)

*mean ± SD; *n*: number; BMI: body mass index; CRP: C-reactive protein

**Fig. 4:**
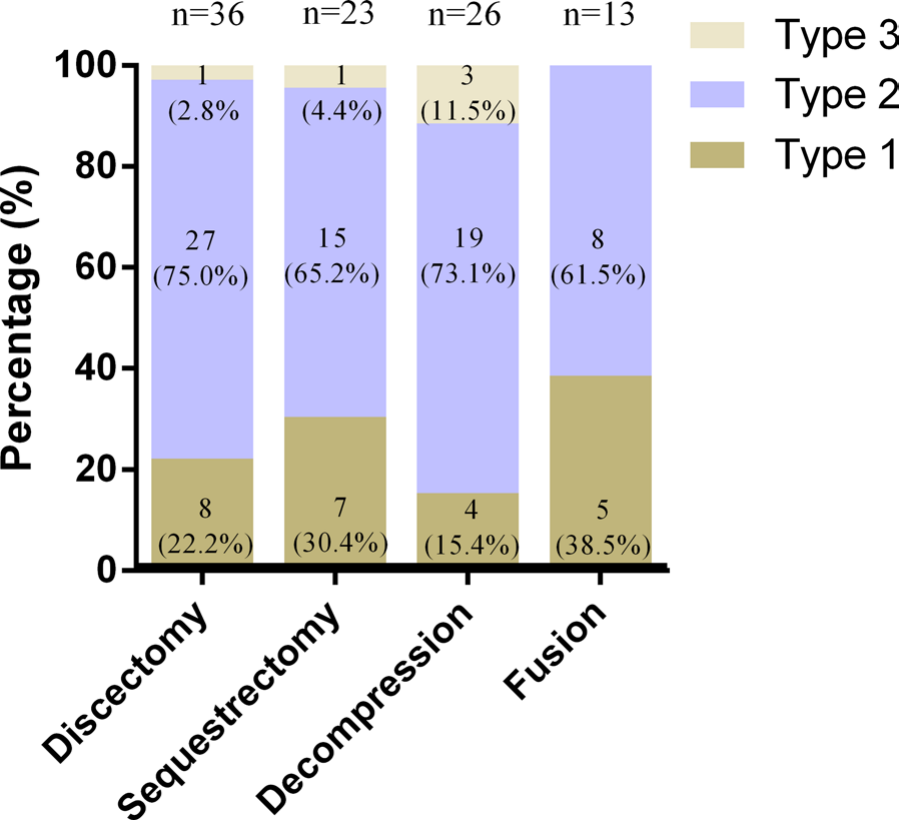
Incidence of new Modic change after lumbar surgical procedures

**Fig. 5:**
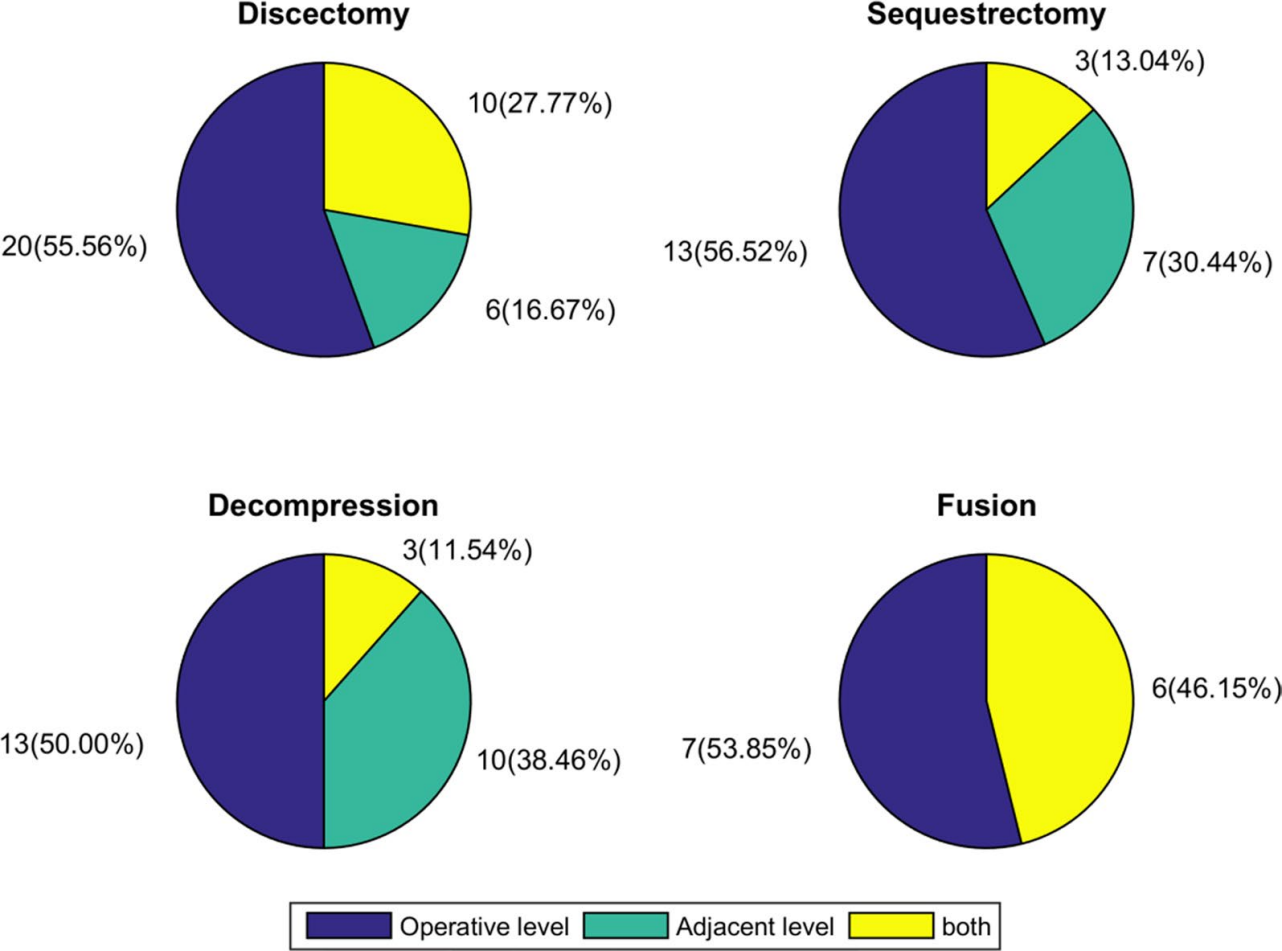
Distribution of affected lumbar levels of new Modic changes after lumbar surgical procedures

The incidence of new MCs, their types, and locations in each group is shown in Figs. 4 and 5. In the microdiscectomy group, new MCs were found in 36 of 93 patients (38.7%), primarily MC2 (75.0%, 27/36). More than half of new MCs were observed at the surgical levels (55.6%, 20/36), followed by at both surgical and adjacent levels (27.8%, 10/36), and at the adjacent levels (16.7%, 6/36).

23 of 60 patients (38.3%) developed new MCs after lumbar microsequestrectomy, 60.9% (14/60) of which were MC2. There were 13 patients (56.5%, 13/23) with new MCs at the surgical levels. New MCs at the adjacent levels were found in seven patients (30.4%, 7/23) at follow-up. The remaining three patients (13.0%, 3/23) had new MCs at both surgical and adjacent levels.

26 of 74 patients (35.2%), who underwent lumbar microdecompression, had new MCs on the postoperative MRI, with MC2 (69.2%, 18/26) being the most common. New MCs were found in 50% of the patients (13/26) at the surgical levels, in 38.5% (10/26) at the adjacent levels, and in 11.5% (3/26) at both surgical and adjacent levels, respectively.

**Fig. 6:**
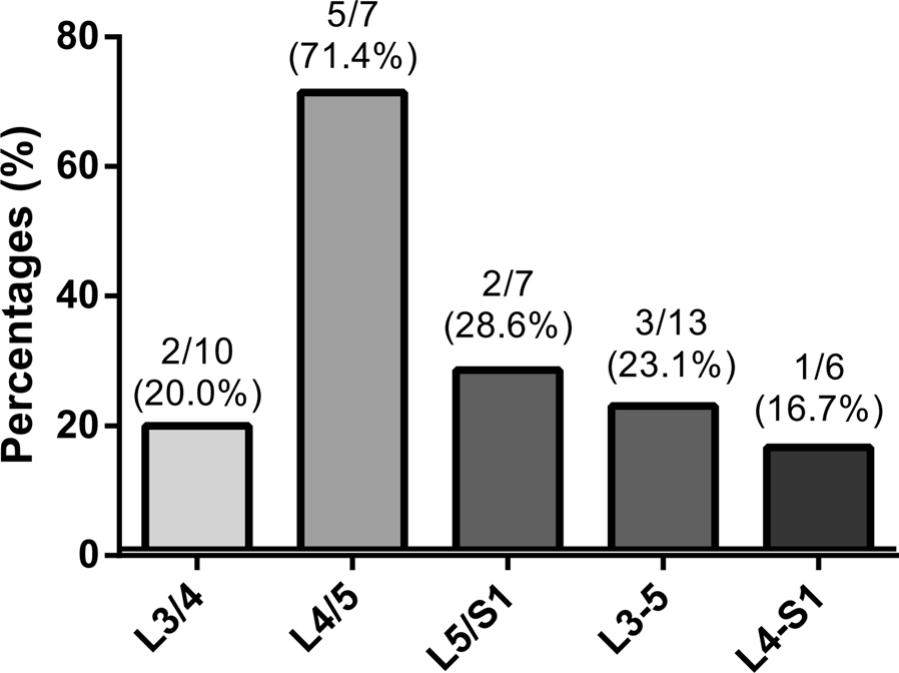
Incidence of new Modic changes in the different fusion levels

There were 43 patients who underwent instrumented TLIF. We didn’t detect the screw loosening and cage subsidence for each fusion cases. Satisfactory bone fusion was obtained in all cases at the final follow-up after TLIF. The evidence of new MCs was detected in 13 patients (30.2%) after lumbar fusion, 7 of which (53.9%, 7/13) occurred at the operative levels and the other 6 (46.2%, 6/13) at both levels (operative and their adjacent segments). 61.5% (8/13) of patients had MC2, followed by MC1 (38.5%, 5/13). None of the patients was found to have MC3 at follow-up. In this study, we also investigated the incidence of new MCs in the different fusion levels. New MCs occurred in 2 of 10 patients (20.0%) with L3/4 fusion, 5 of 7 (71.4%) with L4/5 fusion, 2 of 7 (28.6%) with L5/S1 fusion, 3 of 13 (23.1%) with L3-L5 double-segment fusion, and 1 of 6 (16.7%) with L4-S1 double-segment fusion, indicating that the fusion of L4/5 segment is more likely to develop MCs (Fig. 6).

The pre- and postoperative volumes of MCs in 4 groups are shown in Table 2. In this study, the measured volumes of MCs in pre- and postoperative period represented only operative levels or adjacents levels, not a summation of both. The number of preexisting MCs in lumbar microdiscectomy, microsequestrectomy, microdecompression, and fusion was 33 cases, 21 cases, 32 cases, and 19 cases, respectively. The results indicated that those with preexisting MCs undergoing lumbar microdiscectomy, microsequestrectomy, and microdecompression had significantly larger postoperative volumes than preoperatively (*P* < 0.01). Moreover, the postoperative volumes of MCs in patients who underwent lumbar microdiscectomy, microsequestrectomy, and microdecompression were significantly greater than those before surgery (*P* < 0.01), suggesting that these lumbar surgical procedures can aggravate lesions of the endplates and their adjacent bone marrow. However, no significant difference was detected between pre- and postoperative volumes of MCs in patients with lumbar fusion (*P* > 0.05).

**Table 2:** Pre- and postoperative volumes of Modic changes in the four surgical groups

Groups	Preoperative volume (cm^3^)		Postoperative volume (cm^3^)		*P*
	Preexisting MCs	Preexisting + new MCs	Preexisting MCs	Preexisting + new MCs	
Microdiscectomy	5.1 ± 4.9 (*n* = 33)	3.3 ± 4.6 (*n* = 69)	8.0 ± 6.8 (*n* = 33)	7.0 ± 6.5 (*n* = 69)	< 0.01
Microsequestrectomy	5.4 ± 4.8 (*n* = 21)	3.8 ± 4.7 (*n* = 44)	8.7 ± 7.1 (*n* = 21)	8.1 ± 7.4 (*n* = 44)	< 0.01
Microdecompression	6.2 ± 6.1 (*n* = 32)	4.4 ± 5.9 (*n* = 58)	9.3 ± 6.4 (*n* = 32)	7.7 ± 6.2 (*n* = 58)	< 0.01
Fusion (TLIF)	8.7 ± 7.3 (*n* = 19)	6.6 ± 7.3 (*n* = 32)	7.4 ± 7.5 (*n* = 19)	7.0 ± 7.4 (*n* = 32)	n.s

mean ± SD; n.s., no significance

In summary, patients who underwent lumbar fusion had a tendency towards a lower postoperative incidence rate of new MCs than the other three lumbar surgical procedures.

The incidence and type distribution of new MCs at the different follow-up are shown in Table 3. The cut-off points of follow-up were set for 12 months and 24 months based on the characteristics of the data and the distribution of patients. The occurrence of new MCs could be observed at any phases after lumbar surgeries. Interestingly, the incidence of new MC has shown a downward trend over time. However, postoperative first year seems to be the most active phase for the development of new MCs. Both MC1 and MC2 seem to have the most active phase of change within 12 months, whereas MC3 seems to be a less active situation of bone marrow changes.

**Table 3:** Types distribution of new Modic changes at the different follow-up

Groups	Total incidence	MC1	MC2	MC3
*Microdiscectomy (n = 36)*				
< 12 m	24 (66.6%)	5 (13.9%)	18 (50.0%)	1 (2.8%)
12–24 m	6 (16.7%)	3 (8.3%)	3 (8.3%)	0
> 24 m	6 (16.7%)	0	6 (16.7%)	0
*Microsequestrectomy (n = 23)*				
< 12 m	15 (65.2%)	6 (26.1%)	8 (34.8%)	1 (4.3%)
12–24 m	5 (21.8%)	1 (4.3%)	4 (17.4%)	0
> 24 m	3 (13.0%)	1 (4.3%)	2 (8.7%)	0
*Microdecompression (n = 26)*				
< 12 m	19 (73.1%)	3 (11.5%)	14 (53.8%)	2 (7.7%)
12–24 m	4 (15.4%)	1 (3.9%)	3 (11.5%)	0
> 24 m	3 (11.5%)	0	2 (7.7%)	1 (3.9%)
*Fusion (TLIF) (n = 13)*				
< 12 m	8 (61.5%)	2 (15.4%)	6 (46.1%)	0
12–24 m	2 (15.4%)	1 (7.7%)	1 (7.7%)	0
> 24 m	3 (23.1%)	2 (15.4%)	1 (7.7%)	0

m, months; n, number; MC1, Modic type 1 change; MC2, Modic type 2 change; MC3, Modic type 3 change

## Discussion

### Incidence of new MCs after surgery

Previous studies have mainly focused on investigating the natural course of MCs after a single lumbar surgical procedure. Barzouhi et al. [13] reported that 50.6% (85/168 cases) of patients, who had undergone surgery, developed new MCs in the first year after lumbar discectomy, mostly from no MCs to MC1 (29.8%, 50/168 cases). This has been similarly observed by Barth et al. [14] in the first 2 years after lumbar discectomy. However, the incidence of new MCs in the above studies was higher than those of studies by us and by Rahme et al. [9] during the 3- to 5-year follow-up period after lumbar discectomy. Our study reinforces the strength of the findings from the previous studies, which reported that lumbar discectomy positively accelerates the development of MCs.

However, limited evidence on the course of MCs after lumbar fusion were available. Vital et al. [15] found that in 17 patients with MC1 at baseline, 13 cases progressed to MC2 and 4 converted to normal bone marrow signal at the 6 months follow-up after posterior osteosynthesis. Ohtori et al. [16], who studied 33 cases with MCs, reported, that of the 21 patients with MC1, 10 remained with MC1, 9 exhibited conversion to MC2 and 2 changed to no MCs, and of the 12 patients with MC2, 10 remained with MC2 and 2 converted to no MCs. However, studies on the development of new MCs after lumbar fusion are lacking.

At present, the causes of MCs are still unclear, but endplate microfracture is considered as an important biomechanical factor for the occurrence of MCs [7, 17]. Considering the potential trauma to a disc by lumbar surgery, one hypothesis is that the inflammatory factors and metabolites after disc damage directly permeate into the endplates and vertebral body through the microfracture gaps in the endplates and lead to an inflammatory reaction [18]. Therefore, the finding in this study of more new MCs following lumbar surgical procedures can provide strong evidence for this theory. Besides, a study by Barth et al. [14] reported that higher incidence of MCs was observed in patients with lumbar discectomy compared with lumbar sequestrectomy, indicating that the extent of removal disc material might also be associated with the development of MCs. The intradiscal pressure decrease causing by loss of nucleus material [19] and subsequent change in the pressure load distribution over the disc [20] may contribute to the explanation of this hypothesis. In our study, however, there were no relevant differences in the incidence of new MCs between microdiscectomy and microsequestrectomy patients. The conflicting findings may be attributed to the small sample size of their study and the inconsistent follow-up time of our study.

The published studies only investigated the incidence of new MCs at the surgical levels. This is, at least to our knowledge, the first study to report the incidence of additional new MCs after lumbar surgeries in the adjacent levels of the same patients. In this study, we found that lumbar surgeries seem to promote the development of new MCs both at the surgical and adjacent levels. There were no relevant differences in the incidence of new MCs at the surgical levels between the groups. However, patients with lumbar fusion had a higher risk for the occurrence of new MCs at the adjacent levels than the other three surgical procedures. The rational explanation may be, that the pressure of the adjacent disc and facet joints compensatorily increases due to the loss of mobility in the fused levels, accelerating disc and endplate lesions [21]. In addition, improper selection of pedicle screw insertion sites that damage the endplates and intervertebral discs of adjacent levels may also contribute to the development of new MC at the adjacent level [22]. However, large-sample, multicentre, well-designed studies are still needed to further examine the reliability of the present findings.

Previous studies [1, 23] have reported that MCs mainly occur at the lower two lumbar segments, which may be closely associated with these two lumbar segments where suffered maximum loading. In the present work, we investigated the incidence of new MCs with difference lumbar fusion level, indicating that new MCs occurred much more in patients with L4/5 fusion than in those with other levels fusion. New MCs were specified as the evidence of MCs at the operative level and/or its adjacent segments after surgery in this study. The present work included much more patients who underwent TLIF procedure at L3/4, L4/5, and L5/S1 segments. Therefore, the presence of MCs in their adjacent L4/5 segment after L3/4 and L5/S1 fusion may contribute to the above result. Moreover, the impact of potential biomechanical mechanisms on high incidence of new MCs at L4/5 fusion cannot be ignored either.

Studies on the active phase of MCs after lumbar surgery are limited reporting conflicting results. About 30–40% of patients without preexisting MCs developed new MC1 during the first 3 years and the subsequent progressive increase for MC2 was observed at the 5 years follow-up after lumbar discectomy [6]. In contrast, the result with an increasing prevalence of MC2 during 3–5 years follow-up after lumbar surgery was reported by Rahme et al. [9], being in agreement with the finding in our study. We speculate that it may be associated with the increase in fatty degeneration of the vertebral subchondral bone marrow caused by lumbar surgery [9]. However, a recent new insight tends to consider that the pattern of MCs following lumbar discectomy is complex and not simply increasing [8].

### The volume of MCs based on three-dimensional quantitative analysis

Evidence has suggested a positive correlation between the size of MCs and the extent of low back pain, namely the larger the area of MCs was, the greater the pain of the patient [24, 25]. The semi-quantitative analysis in two dimensions (anteroposterior and/or craniocaudal planes) is still the main method for measuring the area of MCs. Karchevsky et al. [26] divided the vertebral body into 15 small units in the horizontal plane and evaluated the morphologiy of reactive endplate marrow changes by summing up the involvement of each small unit. According to the standardized evaluation classification proposed by the Nordic Modic Consensus Group, Jensen et al. [27] classified the area of MCs based on its relative depth of extension of the vertebral body height as four categories: endplate only, < 25%, 25–50% and > 50%. However, the above methods that only calculated the involvement units of MCs in the horizontal or vertical division are inadequate. Considering that the limitation of the above measuring methods, Kuisma et al. [23] introduced a method that combined the maximum area of MCs by cutting the vertebral body into four small units in the horizontal plane in the axial T1-weight images with the maximum vertical depth of MC in the sagittal T2-weight images. Subsequently, a study by Hanmolu et al. [28] used a classification that divided the vertebral body into four equal sections across the anteroposterior and craniocaudal planes (16 small units) to evaluate the relationship between the area of MC1 and Oswestry disability index (ODI), reporting that the MC1 involvement extent was significantly associated with changes in the ODI.

However, MCs usually have irregular shapes in three-dimension. The qualitative or quantitative analysis in two dimensions for the sizes of MCs will inevitably lead to measurement errors, resulting in unreliable results. Previous studies [9, 10, 13] have been reported that the majority of patients with MC1 and MC2 progressed in sizes following lumbar discectomy. To our best knowledge, this study for the first time investigates the effects of four lumbar surgical procedures on the volumes of MCs using quantitative analysis in three dimension. The current study found that patients with preexisting MCs have an increase in the volumes of endplate lesions after surgery. However, postoperative volume changes of MCs were not significant for patients who underwent lumbar fusion, indicating that lumbar fusion may be an appropriate procedure for patients with preexisting MCs. We believe that the following two potential reasons may be able to respond to this finding: (1) posterior decompression surgery disrupts the soft tissue and bony tissue behind the lumbar spine, which may cause biomechanical changes in the lumbar spine, triggering the development of new MCs or exacerbating preexisting Modic endplate lesions; (2) lumbar fusion not only can provide immediate spinal stability, it also has a certain effect on the reconstruction of the sagittal balance of the spine, which can delay or even hinder the further development of MCs in the biomechanical level.

### Limitations

As with other studies, this study also suffers several limitations. The main limitation to be considered is that the follow-up between study populations varied greatly due to the characteristic of the retrospective study raising the difficulty of investigating the natural course of MCs following lumbar surgery. Furthermore, this study mainly focused on specific patients who underwent lumbar surgery. The results of this study therefore can only be generalized to patients with symptomatic lumbar degenerative diseases. Additionally, we did not rule out other potential factors reported in the published articles, which might affect the development of MCs such as conservative treatment. [29]. Finally, the findings of this study with the small sample size (especially when comparing four different surgical procedures) need to be further examined by a larger sample and multicentre studies.

## Conclusions

This retrospective study shows that lumbar surgery was associated with the development of new MCs and progression of preexisting MCs. Lumbar fusion appears to be affected to a lesser extent compared to non-fusion surgery. Following surgery, most patients developed MC2 at the surgical levels and both MC1 and MC2 seem to have the most active phase of change within 12 months, whereas MC3 seems to be a less active situation of bone marrow changes.

## Data Availability

The datasets generated and analysed during the current study are available from the corresponding author on reasonable request.

## Abbreviations

MRI: Magnetic resonance imaging
MCs: Modic changes
DD: Disc degeneration
ULBD: Unilateral laminotomy for bilateral decompression
TLIF: Transforaminal lumbar interbody fusion
T1WI: T1-weighted images
ST: Slice thickness
TR: Time of repetition
TE: Time to echo
FOV: Field of view
T2WI: T2-weighted images
MC1: Modic type 1 change
MC2: Modic type 2 change
MC3: Modic type 3 change
